# Aortic and biventricular function in pediatric meningococcal septic shock survivors as assessed with MRI

**DOI:** 10.1186/1532-429X-14-S1-P199

**Published:** 2012-02-01

**Authors:** Heynric Grotenhuis, Hennie Knoester, Jos J Westenberg, Lucia JM Kroft, Albert de Roos

**Affiliations:** 1 Radiology, Leiden University Medical Center, Leiden, Netherlands; 2 Pediatric Intensive Care, Emma Childrens Hospital AMC, Amsterdam, Netherlands

## Summary

Septic shock is one of the major causes of death in children and is characterized by a massive inflammatory response. To present date, no studies have been performed to assess the impact of such an ‘inflammatory hit’ on aortic wall structure and myocardial performance.

This study shows that despite adequately preserved systolic biventricular function, frequently reduced aortic elasticity may indicate aortic wall pathology, being associated with biventricular hypertrophy and concomitant delayed biventricular relaxation in pediatric patients after septic shock.

## Background

Septic shock is one of the major causes of death in children and is characterized by a massive inflammatory response. To present date, no studies have been performed to assess the impact of such an ‘inflammatory hit’ on aortic wall structure and myocardial performance. The objectives of the current study were therefore to prospectively assess aortic elasticity and biventricular systolic and diastolic function in a group of pediatric patients after meningococcal septic shock by using MRI.

## Methods

Eighteen pediatric meningococcal septic shock survivors (8 male; mean age±standard deviation 14.5years±3.9; imaging performed 8.2years±2.4 after septic shock) treated with at least 2 inotropic and vasoconstrictive agents for 48 hours or longer and 18 for age and gender matched controls were prospectively studied. Routine MRI was used to assess aortic pulse wave velocity (PWV) and systolic and diastolic biventricular function. Independent-samples-t-test and Pearson-correlation-coefficient were used for statistical analysis.

## Results

Sepsis patients showed reduced aortic elasticity vs. controls (PWV in aortic arch: 4.1m/s±0.3 vs. 3.3m/s±0.5, P<0.01; PWV in descending aorta: 3.9m/s±0.9 vs. 3.2m/s±0.4, P<0.01). Systolic biventricular function was preserved (LV ejection fraction 57%±8 vs. 56%±6, P=0.74; RV ejection fraction 56%±8 vs. 52%±6, P<0.01), whereas biventricular mass was increased (LV 52.1gram/m2±8.4 vs. 36.0gram/m2±9.9, P<0.01; RV 26.8gram/m2±6.5 vs. 10.4gram/m2±5.0, P<0.01). Also, delayed biventricular relaxation was found after sepsis: peak filling rates corrected for end-diastolic-volume (PFREDV) across the mitral and tricuspid valve were significantly reduced (mitral: PFREDV of E wave 2.54±0.56 vs. 3.08±0.63, P=0.01; PFREDV of A wave 1.10±0.26 vs. 1.31±0.30, P=0.03; tricuspid: PFREDV of E wave 1.81±0.44 vs. 2.09±0.29, P=0.04; PFREDV of A wave 1.11±0.22 vs. 1.42±0.39, P<0.01). Increased PWV in aortic arch and descending aorta were associated with increased LV mass (r=0.62, P<0.01, and r=0.51, P<0.01, respectively) and delayed LV relaxation parameters.

## Conclusions

Despite adequately preserved systolic biventricular function, reduced aortic elasticity in pediatric patients after septic shock may indicate aortic wall pathology, being associated with biventricular hypertrophy and concomitant delayed biventricular relaxation. Long-term prognosis after septic shock may therefore be adversely affected considering the cumulative effects of cardiovascular disease during a lifetime.

## Funding

No disclosures.

